# Editorial: New rootstocks for fruit crops: breeding programs, current use, future potential, challenges and alternative strategies, volume II

**DOI:** 10.3389/fpls.2025.1718492

**Published:** 2025-10-23

**Authors:** Sergio Ruffo Roberto, Vittorino Novello, Gennaro Fazio

**Affiliations:** ^1^ Department of Agronomy, State University of Londrina, Londrina, Brazil; ^2^ Dipartimento di Scienze Agrarie, Forestali ed Alimentari, University of Turin, Turin, Italy; ^3^ Plant Genetic Resources Unit, United States Department of Agriculture/Agricultural Research Service (USDA/ARS), Geneva, NY, United States

**Keywords:** rootstocks, fruit, breeding, scion compatibility, micropropagation

## Introduction

Rootstocks represent a critical component in the physiology and management of fruit crops, exerting profound effects on scion growth, productivity, and resilience. By mediating the uptake and translocation of water, minerals, and signaling molecules, rootstocks influence phenotypic traits such as vigor, canopy architecture, and fruit set. Moreover, they determine the capacity of fruit crops to adapt to heterogeneous soil and climatic conditions, making them indispensable for the long-term sustainability of orchard systems (Ling et al., 2025).

A principal advantage of rootstock utilization lies in their ability to regulate tree size and optimize orchard design. Dwarfing and semi-dwarfing rootstocks, for example, enable high-density planting systems that increase land-use efficiency, facilitate mechanization, and improve labor productivity. Additionally, rootstocks can stabilize growth patterns, enhance uniformity across orchards, and contribute to the consistency of fruit quality traits, including size, firmness, and biochemical composition. Such effects are often attributed to rootstock–scion interactions that modulate hormonal balance, carbohydrate partitioning, and nutrient dynamics (Domingues et al., 2021).

Beyond horticultural traits, rootstocks are a vital source of genetic resistance to soilborne pathogens and pests, including nematodes, fungi, and viruses. Their defensive role significantly reduces the dependence on chemical inputs, aligning fruit production with integrated pest management (IPM) and sustainable agriculture principles. Furthermore, rootstock-mediated tolerance to abiotic stresses—such as salinity, drought, and low nutrient availability—extends the geographical range of fruit cultivation and mitigates yield losses under suboptimal environmental conditions (Mao et al., 2022).

Breeding programs have increasingly focused on the genetic improvement of rootstocks to address emerging challenges in fruit production. Advances in molecular genetics, quantitative trait locus (QTL) mapping, and marker-assisted selection (MAS) facilitate the development of rootstocks with enhanced biotic and abiotic stress resistance, greater nutrient-use efficiency, and superior graft compatibility. More recently, genomic selection and biotechnological approaches, including transgrafting and genome editing, have further accelerated rootstock innovation. These strategies collectively strengthen the capacity of fruit crops to maintain high yield potential, resilience, and quality in the face of climate change and evolving pest and disease pressures (Harrison et al., 2016).

The aim of this Research Topic was to present the latest results of new rootstocks developed using classic and modern selection techniques and forecast novel applications.

In this context, Alonso-Forn et al. investigated the role of root morphology and anatomy in grapevine rootstocks’ tolerance to water deficit and recovery. Thirteen rootstock genotypes, including commercial and newly bred varieties, were evaluated under controlled conditions over two seasons. The research revealed that rootstocks with higher root length density (RLD) and fine roots maintained better water status during severe drought, while smaller xylem vessel diameters improved water transport efficiency and recovery. A trade-off was observed between root density (enhancing water uptake) and transport efficiency. Rootstocks like ‘420A’, ‘41B’, ‘RM2’, and ‘Fercal’ demonstrated superior drought resilience, while the RG-series showed no significant advantage over established genotypes. These findings highlight the importance of root traits in drought tolerance and suggest their potential use in breeding programs, though further field validation is needed.

Lawrence et al. investigated the long-term interactions between 19 rootstocks (from Budagovsky, Geneva, and Malling series) and five apple cultivars (‘Empire’, ‘Gala’, ‘Honeycrisp’, ‘Mutsu’, and ‘Delicious’) in two orchards in Western New York, US. The research highlighted that rootstock effects on tree survival, growth, yield, and yield efficiency significantly vary depending on the cultivar, while fruit quality traits like size, firmness, and soluble solids showed minimal interaction. These new findings emphasize the importance of selecting specific rootstock-cultivar combinations to optimize orchard performance and profitability, tailored to regional growing conditions.

Moving toward another important tree crop species, i.e., citrus (*Citrus sinensis* L.), Devite et al. evaluated the performance of ‘Valencia’ sweet orange grafted onto three dwarfing citrandarin rootstocks (‘IAC 1600’, ‘IAC 1697’, and ‘IAC 1711’) compared to ‘Swingle’ citrumelo under high-density planting conditions. The research focused on productivity, water-use efficiency, vegetative growth, and Huanglongbing (HLB) incidence. ‘Swingle’ citrumelo demonstrated high per-tree yield and canopy volume but lower water-use efficiency and higher HLB susceptibility. In contrast, citrandarins, particularly ‘IAC 1600’, exhibited superior water-use efficiency, drought tolerance, and lower HLB incidence, making them better suited for water-limited and HLB-endemic regions. The findings highlighted the importance of rootstock selection in optimizing citrus productivity and sustainability under varying environmental conditions. Within the same species, Febres et al. investigated graft incompatibility in citrus, focusing on the rootstock ‘US-1283,’ a citrandarin hybrid, which exhibits incompatibility with ‘Bearss’ lemon and ‘Valencia’ sweet orange, manifesting as stem grooving and necrosis. Using transcriptome analysis, the researchers compared compatible (‘US-812)’ and incompatible (‘US-1283’) graft combinations to identify molecular mechanisms underlying incompatibility. Results revealed significant transcriptional reprogramming in the incompatible rootstock, particularly below the graft union, with differentially expressed genes (DEGs) linked to oxidative stress, plant defense, and lignin biosynthesis. The findings suggest that incompatibility may arise from signaling miscommunications between the scion and rootstock, potentially involving danger-associated molecular patterns (DAMPs). This study provided insights into graft incompatibility mechanisms and highlights potential targets for improving compatibility in citrus breeding.

Some interesting aspects of vegetable speed breeding were investigated by He et al. The review article described the use of speed breeding (SB) technology in plant factories to address the challenges of lengthy breeding cycles for vegetable crops. SB, which optimizes environmental factors like light, temperature, CO_2_, and nutrients, significantly accelerates plant growth and generation turnover. When integrated with advanced breeding technologies such as genome editing and high-throughput genotyping, SB in plant factories emerges as a promising platform for developing high-yield, resilient vegetable varieties efficiently. The review highlighted the opportunities, challenges, and potential of this approach to enhance crop breeding precision and efficiency.

Finally, Traini et al. assessed micro propagated, grafted on not suckering rootstock and own-rooted plants by layering from three Italian hazelnut (*Corylus avellana* L.) cultivars. Authors found that the micro propagated plants, regardless of the variety considered, even being smaller than the other plants at the beginning of the plantation, reached similar sizes as the other plants after four growing seasons. In addition, it was described that micro propagated plants exhibited greater uniformity in growth compared to grafted ones, while own-rooted plants displayed more variability, and no significant differences in yield performance and canopy volume were observed among the three propagation methods.
